# Sequelae in Adults at 6 Months After COVID-19 Infection

**DOI:** 10.1001/jamanetworkopen.2021.0830

**Published:** 2021-02-19

**Authors:** Jennifer K. Logue, Nicholas M. Franko, Denise J. McCulloch, Dylan McDonald, Ariana Magedson, Caitlin R. Wolf, Helen Y. Chu

**Affiliations:** 1 Division of Allergy and Infectious Diseases, Department of Medicine, University of Washington, Seattle

## Abstract

This cohort study analyzed persistent symptoms among adults with coronavirus disease 2019 up to 9 months after illness onset.

## Introduction

Many individuals experience persistent symptoms and a decline in health-related quality of life (HRQoL) after coronavirus disease 2019 (COVID-19) illness.^1^ Existing studies have focused on hospitalized individuals 30 to 90 days after illness onset^2,3,4^ and have reported symptoms up to 110 days after illness.^3^ Longer-term sequelae in outpatients have not been well characterized.

## Methods

A longitudinal prospective cohort of adults with laboratory-confirmed severe acute respiratory syndrome coronavirus 2 (SARS-CoV-2) infection was enrolled at the University of Washington with a concurrent cohort of healthy patients in a control group (eAppendix in the Supplement). Electronic informed consent was obtained, and the study was approved by the University of Washington human participants institutional review board. This study followed the Strengthening the Reporting of Observational Studies in Epidemiology (STROBE) reporting guideline. COVID-19 symptom data were obtained at the time of acute illness or retrospectively recounted at a 30-day enrollment visit. A total of 234 participants with COVID-19 were contacted between August and November 2020 to complete a single follow-up questionnaire between 3 and 9 months after illness onset. We did not perform statistical tests for this descriptive analysis because of the small numbers in each subgroup. Data analysis was conducted in R version 4.0.2 (R Project for Statistical Computing).

## Results

A total of 177 of 234 participants (75.6%; mean [range] age, 48.0 [18-94] years; 101 [57.1%] women) with COVID-19 completed the survey. Overall, 11 (6.2%) were asymptomatic, 150 (84.7%) were outpatients with mild illness, and 16 (9.0%) had moderate or severe disease requiring hospitalization (Table). Hypertension was the most common comorbidity (23 [13.0%]). The follow-up survey was completed a median (range) of 169 (31-300) days after illness onset among participants with COVID-19 (Figure, A) and 87 (71-144) days after enrollment among 21 patients in the control group. Among participants with COVID-19, persistent symptoms were reported by 17 of 64 patients (26.6%) aged 18 to 39 years, 25 of 83 patients (30.1%) aged 40 to 64 years, and 13 of 30 patients (43.3%) aged 65 years and older. Overall, 49 of 150 outpatients (32.7%), 5 of 16 hospitalized patients (31.3%), and 1 of 21 healthy participants (4.8%) in the control group reported at least 1 persistent symptom. Of 31 patients with hypertension or diabetes, 11 (35.5%) experienced ongoing symptoms.

**Table.** Demographic and Clinical Characteristics of the Study Cohort

Characteristic	No. (%)				
	Total recovered individuals (n = 177)	Inpatients (n = 16)	Outpatients (n = 150)	Asymptomatic individuals (n = 11)	Healthy controls (n = 21)
Age, mean (SD), y	48.0 (15.2)	54 (15.1)	46.3 (14.3)	63.8 (18.8)	50.8 (15.8)
Sex					
Women	101 (57.1)	8 (50.0)	87 (58.0)	6 (54.5)	11 (52.4)
Men	76 (42.9)	8 (50.0)	63 (42.0)	5 (45.5)	10 (47.6)
BMI, mean (SD)	27.1 (5.8)	28.7 (9.1)	26.4 (6.6)	26.3 (5.4)	25.2 (7.1)
Race/ethnicity					
Non-Hispanic/Latino					
White	135 (76.3)	6 (37.5)	121 (80.7)	8 (72.7)	16 (76.2)
Black	3 (1.7)	1 (6.2)	2 (1.3)	0	0
Other^a^	31 (17.5)	8 (50.0)	21 (14.0)	2 (18.2)	5 (23.8)
Hispanic/Latino	7 (4.0)	1 (6.2)	5 (3.3)	1 (9.1)	0
Missing	1 (0.6)	0	1 (0.7)	0	0
Influenza vaccination	130 (73.4)	12 (75.0)	109 (72.7)	9 (81.8)	18 (85.7)
Comorbidities					
Hypertension	23 (13.0)	3 (18.8)	18 (12.0)	2 (18.2)	0
Diabetes	9 (5.1)	4 (25.0)	4 (2.7)	1 (9.1)	1 (4.8)
Active smoking	8 (4.5)	0	7 (4.7)	1 (9.1)	1 (4.8)
Highest level of care accessed during acute illness					
None	107 (60.5)	0	96 (64.0)	11 (100)	21 (100)
Primary care	37 (20.9)	0	37 (24.7)	0	0
Urgent room or emergency department	17 (9.6)	0	17 (11.3)	0	0
Admitted to hospital or ICU	16 (9.0)	16 (100)	0	0	0
Post–COVID-19 follow-up characteristics					
Time after illness onset, median (SD), d^b^	169 (39.5)	179 (44.9)	169 (37.1)	139 (47.1)	87 (31.3)
Persistent symptoms^c^					
0	119 (67.2)	10 (62.5)	98 (65.3)	11 (100.0)	20 (95.2)
1-2	29 (16.4)	2 (12.5)	28 (18.7)	0	0
≥3	24 (13.6)	3 (18.8)	21 (14.0)	0	1 (4.8)
Missing	7 (4.0)	1 (6.3)	3 (2.0)	0	0
Worsened quality of life^d^	53 (29.9)	7 (43.8)	44 (29.3)	2 (18.2)	2 (1.4)

Abbreviations: BMI, body mass index (calculated as weight in kilograms divided by height in meters squared); COVID-19, coronavirus disease 2019; ICU, intensive care unit.

^a^ Other race/ethnicity included American Indian or Alaska Native, Asian, Native Hawaiian or other Pacific Islander, and more than 1 race.

^b^ Time since symptom onset in severe/mild cohorts, time since first positive test in asymptomatic individuals, time since enrollment in healthy controls.

^c^ Participants with COVID-19 were asked whether they experienced continued symptoms from their COVID-19 illness. Healthy patients in the control group were asked whether they experienced symptoms from an illness at the time of follow up survey completion.

^d^ Quality of life was assessed using a sliding scale ranging from 0 (worst imaginable health) to 100 (best imaginable health). Worsened quality of life was defined as a 10-point decrease in health status from before COVID-19 to the time of survey completion.

**Figure.**
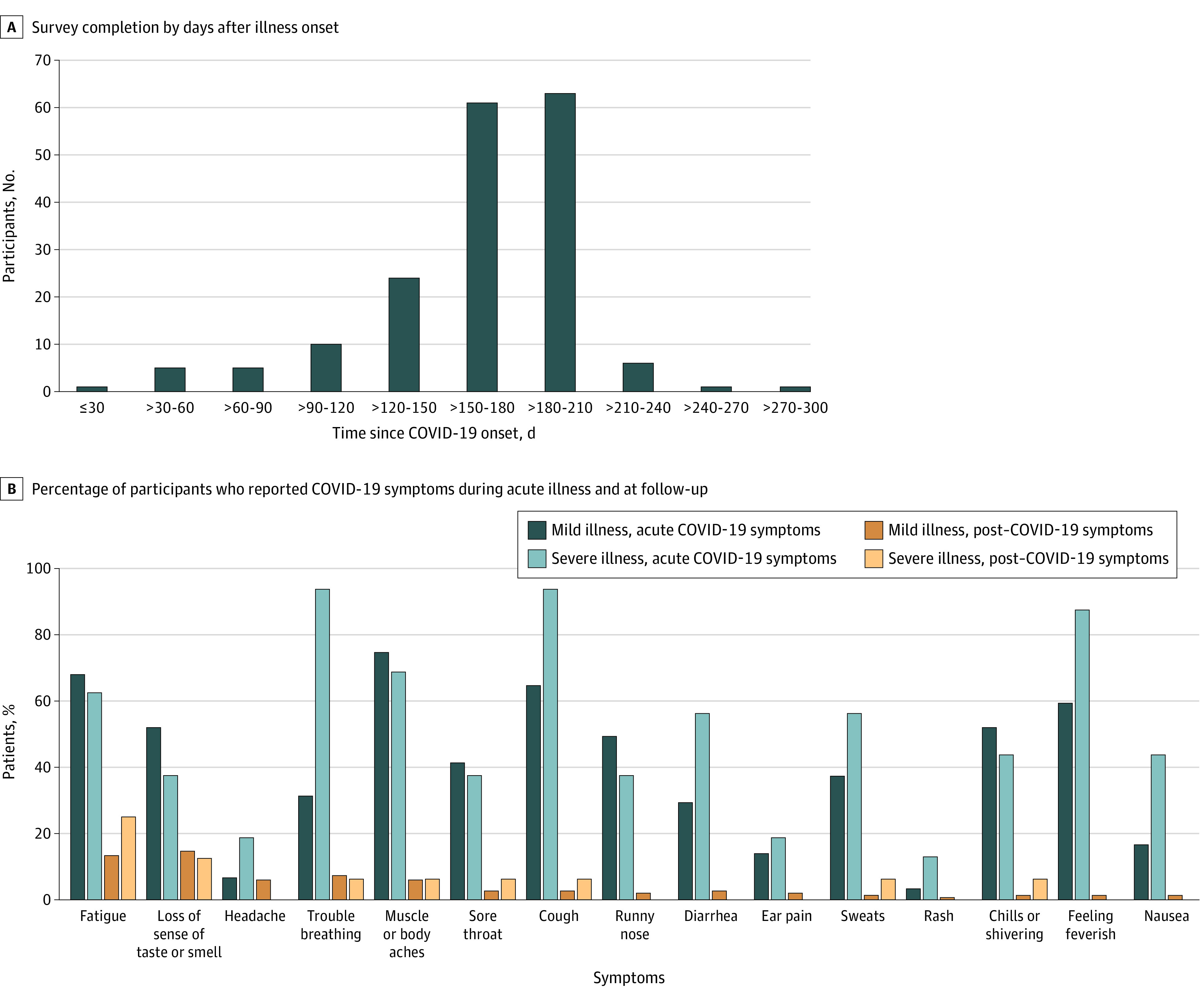
Time of Survey Completion and Coronavirus Disease 2019 (COVID-19) Symptoms

The most common persistent symptoms were fatigue (24 of 177 patients [13.6%]) and loss of sense of smell or taste (24 patients [13.6%]) (Figure, B). Overall, 23 patients (13.0%) reported other symptoms, including brain fog (4 [2.3%]). A total of 51 outpatients and hospitalized patients (30.7%) reported worse HRQoL compared with baseline vs 4 healthy participants and asymptomatic patients (12.5%); 14 patients (7.9%) reported negative impacts on at least 1 activity of daily living (ADL), the most common being household chores.

## Discussion

In this cohort of individuals with COVID-19 who were followed up for as long as 9 months after illness, approximately 30% reported persistent symptoms. A unique aspect of our cohort is the high proportion of outpatients with mild disease. Persistent symptoms were reported by one-third of outpatients in our study, consistent with a previously reported study,^4^ in which 36% of outpatients had not returned to baseline health by 14 to 21 days following infection. However, this has not been previously described 9 months after infection.

Consistent with existing literature, fatigue was the most commonly reported symptom.^2,3,4^ This occurred in 14% of individuals in this study, lower than the 53% to 71%^2,3,4^ reported in cohorts of hospitalized patients, likely reflecting the lower acuity of illness in our cohort. Furthermore, impairment in HRQoL has previously been reported among hospitalized patients who have recovered from COVID-19; we found 29% of outpatients reported worsened HRQoL.^5^

Notably, 14 participants, including 9 nonhospitalized individuals, reported negative impacts on ADLs after infection. With 57.8 million cases worldwide, even a small incidence of long-term debility could have enormous health and economic consequences.^6^

Study limitations include a small sample size, single study location, potential bias from self-reported symptoms during illness episode, and loss to follow-up of 57 participants. To our knowledge, this study presents the longest follow-up symptom assessment after COVID-19 infection. Our research indicates that the health consequences of COVID-19 extend far beyond acute infection, even among those who experience mild illness. Comprehensive long-term investigation will be necessary to fully understand the impact of this evolving viral pathogen.

## Notes

Supplement. **eAppendix.** Supplemental Methods
