# Error in Table

**DOI:** 10.1001/jamanetworkopen.2021.4572

**Published:** 2021-03-09

In the Research Letter titled “Sequelae in Adults at 6 Months After COVID-19 Infection,”^1^ published February 19, 2021, there was an error in the Table. The percentage of patients in the healthy control group who reported worsened quality of life should have read 9.5. This article has been corrected.^1^
