# Supplementary material for: Sequelae in Adults at 6 Months After COVID-19 Infection
Source: JAMA Netw Open. 2021 Feb 19;4(2):e210830. doi: 10.1001/jamanetworkopen.2021.0830 (PMC7896197; doi:10.1001/jamanetworkopen.2021.0830)
Supplement: Supplement. — eAppendix. Supplemental Methods [file jamanetwopen-e210830-s001.pdf]

Supplemental Online Content

Logue JK, Franko NM, McCulloch DJ, et al. Sequelae in adults at 6 months after COVID-19 infection. *JAMA Netw Open*. 2021;4(2):e210830. doi:10.1001/jamanetworkopen.2021.0830

eAppendix. Supplemental Methods

This supplemental material has been provided by the authors to give readers additional information about their work.

© 2021 Logue JK et al. *JAMA Network Open.*

eAppendix. Supplemental Methods

Study design
This was a prospective cohort study and follows the Strengthening the Reporting of Observational Studies in Epidemiology (STROBE) reporting guideline. Participants with COVID-19 were enrolled from February 2020 through November 2020 into a prospective longitudinal immune profiling study; data for the present sub-study were obtained from this cohort.

Study population
Individuals with laboratory-confirmed SARS-CoV-2 infection were recruited from two groups. First, inpatients hospitalized at Harborview Medical Center, University of Washington Medical Center, or Northwest Hospital in Seattle, WA, were identified through a laboratory alert system. Second, outpatients receiving care at University of Washington clinics or testing positive through the Seattle Flu Study were invited to participate. A subset of outpatients were asymptomatic at the time of diagnosis, and confirmed to be symptom-free for the first 30 days after diagnosis. Healthy controls were recruited through email and flyer advertisements. Individuals 18 years or older were eligible for inclusion. Exclusion criteria were pregnancy and weight under 110 pounds.

The planned sample size for follow-up data collection was approximately 230 participants, including 200 COVID-19 positive participants and 30 healthy controls. Two hundred and seventy-seven individuals were invited to participate, and 198 completed surveys. These numbers reflected the number of participants already enrolled in an ongoing COVID-19 prospective cohort study.

Study procedures
Participants or their legally authorized representatives completed electronic informed consent. Sociodemographic and clinical data were collected from electronic chart review and from participants via a data collection questionnaire at the time of enrollment. The questionnaire collected data on the nature and duration of symptoms, medical comorbidities, and care-seeking behavior. Asymptomatic individuals were identified based on responses to this symptom questionnaire. Participants with symptoms but who remained outpatient were considered to have mild illness, and all hospitalized participants were categorized as having moderate/severe illness.

Inpatients were enrolled during their hospital admission. After hospital discharge, these participants were asked to present to an outpatient clinical research site approximately 30 days after symptom onset for follow-up. Outpatients and asymptomatic individuals completed their enrollment, data collection questionnaire, and biological sample collection at an outpatient visit at approximately 30 days after symptom onset (or positive test, for asymptomatic individuals). Only data from the enrollment and electronic follow-up questionnaire were used in this publication. Healthy controls were matched by age, sex, and race, and completed biological sample collection on the same schedule as the COVID-19 cohort.

Between 3-9 months after illness onset, participants were asked to complete an electronic follow-up questionnaire regarding persistent symptoms, additional medical care, changes in quality of life, and impact on activities of daily living (ADLs). The survey was sent electronically to capture as many participants as possible, due to limitations of in-person appointments during the pandemic. Participants filled out this questionnaire once, irrespective of time post-illness. Of the 234 Covid-19 participants contacted, 57 (24.4%) did not respond. Those who did not respond were younger, predominantly male, and 89% had experienced mild illness compared to 85% of those who responded.

Data analysis
Data were analyzed in R version 4.0.2. Mean and standard deviation were calculated for continuous variables, and percentages were calculated for categorical variables.

Human subjects
This study was approved by the University of Washington Human Subjects Institutional Review Board.

Word Count = 519

© 2021 Logue JK et al. *JAMA Network Open.*
